# Collectanea Medica: Consisting of Anecdotes, Facts, Extracts, Illustrations, &c.

**Published:** 1821-11

**Authors:** 


					COLLECTANEA MEDIC A:
CONSISTING OF
ANECDOTES, FACTS, EXTRACTS, ILLUSTRATIONS, &c.
Relating to the History or the Art of Medicine, and the
Auxiliary Sciences.
Floriferis ut apes in saltibus omnia libant,
Omnia nos itidein dcpascimur aurea dicta.
Continuation of our Abstract of Baron Humboldt's Dissertation on
Isothermal Lines, and the Distribution of Heat over the Globe.
HAVING considered the division of heat between winter and
summer on the same isothermal line, wc shall now point out the
numerical ratios between the mean temperature of spring and winter,
and between that of the whole year and the warmest month. From
the parallel of Rome to that of Stockholm, and consequently between
the isothermal lines of 60*8? and 41?, the difference of .the months of
April and May is every where 10-8 or 12*6, and all the successive
months are those which present the most rapid increase of tempera-
ture. But, as in northern countries, (in Sweden, for example,) the
month of April is only 37*4, the 10*8 or 12*6' which the month of
1
Baron Humboldt on Isothermal Lines. 4G9
May adds,* necessarily produces there a much greater cffcct on the
development of vegetation than in the south of Europe, where the
mean temperature of April is from 53'6 to 55*4. It is from an ana-
logous cause that, in passing from the shade to the sun, cither in our
climates in winter, or between the tropics on the back of the Cordil-
leras, we are more affected by the difference of temperature than in
summer and in the plains; though in both cases the thermomctrical
difference is the same,?for example, from 5*4 to 7*2. Near the
polar circle, the increase of the vernal heat is not only more sensible,
but it extends equally to the month of June. At Dronthcim, the
temperatures of April and May, like those of May and June, differ not
10*8 or 12*6, but 14*4 or l6*2.
-In distinguishing upon the same isothermal line the places which
approach its concave or convex summits, in the same system of climates
in the northern and southern regions, we shall find,?
1st. That the increase of the vernal temperature is great, (from
14-4 or 16-2, in the space of a month,) and equally prolonged, whcrc-
ever the division of the annual heat between the seasons is very un-
equal, as in the north of Europe, and in the temperate part of the
United States.
2dly. That the vernal increase is great, (at least above 9? or 10*8,)
but little prolonged, in the temperate part of Europe.
3dly. That the increase of the vernal temperature is small, (scarcely
7*2,) and equally prolonged, wherever there is an insular climate.
4thly. That in every system of climates, in the zones contained be-
tween the same meridians, the vernal increase is smaller, and less
equally prolonged, in low than in high latitudes.
The isothermal zone from 53-6 to 55*4 may serve as an example for
confirming these different modifications of spring. In Eastern Asia,
near the concave summit, the differences of temperature between the
four months of March, April, May, and June, are very great, and
very equal, (15*7, 13*3, and 13*9.) In advancing westward towards
Europe, the isothermal line rises again; and, in the interior of the
country, near the convex summit, the increase is still greater, but little
prolonged: that is to say, that, of the four months which succeed one
another, there arc only two whose difference rises to 13?; they arc
9'4, 13-3, 4-1. Farther west, on the coasts, the differences become
small and equal,?viz. 3'6, 6*5, 5-6. In crossing the Atlantic, we
approach the western concavc summit of the isothermal line of 53'6.
?The increase of vernal temperature shows itself anew, and almost as
great and as much prolonged, as near the Arctic concave summit.
The differences of the four months are 10*4, 13*9) an(I 10*8. In the
curve of annual temperature, the spring and autumn mark the transi-
tions from the minimum and the maximum. The increments arc na-
turally slower near the summits than in the intermediate part of the
curve. Here they are greater, and of longer continuance, in propor-
* In calculating for Europe, from 46? to 48? ot lat. tor ten years, the mean
temperatures of every ten days, we find that the decades which succeed one an-
other differ near the summits of the annual curve only 1*4-1, while the differences
rise in autumn lioui 3'6 to 5*4, and in spring from 5*4 to 7-2.
470 Collectanea Medic a.
lion to the differcncc of the extreme ordinates. The autumnal decrease
of temperature is less rapid than the vernal increase, because the sur-
face of the earth acquires the maximum of heat slower than the
atmosphere; and because, in spite of the serenity of the air which
prevails in autumn, the earth loses slowly, by radiation, the heat which
it has acquired. The following Table will show how uniform the
laws arc which J hare just established:
Names of Places.
I. Group,?Concave Summits in America
Natchez
Williamsburg
Cincinnati. ? ?
Philadelphia*
New York: ?
Cambridge ?
Quebec
Nain
II. Group,?Convex Summits in EtlTope,
1. Continental Climate:
Rome?
Milan
Geneva
Bnda
Paris ?...
Gottiugen   ? ?
JU psal
Petersburg
Umeo
Ulco
Enontekies
2. Climate of the Coast:
Nantes
London
Dublin
Edinburgh
North Cape
III. Group,?Concave Summit of Asia.
Pekin
Lat.
31-28?
37*18
39* 0
39*56
40*40
42*25
46-47
57- 0
4153
45*28
46*12
47-29
48-50
51-32
59*51
59-56
63*50
65* 0
68-30
47*13
51*30
53*21
55*57
71* 0
39-54
March.
57*90
46-4
43*7
44*1
38*7
34-5
23-0
6*8
50-4
47-8
39-6
38*3
4S-3
34*2
29*5
.27-5
23*0
14"0
11*5
50*0
44*2
41*9
41'4
25*0
41*4
April.
66-2?
61*2
57*4
53*6
49-1
45*5
39*6
27*5
55*4
51*1
45*5
49'1
48*2
44*2
39*7
37*0-
34*2
26-2
26*6
53*6
49-8
45*3
47 3
30-0
57-0
May.
72-7=
66-6
61*2
62*1
65*8
56*8
54*7
37*0
66-9
65" L
58*1
64*8
60-1
57 *7
48*7
50-2
43*7
41*0
36*5
60-1
56*5
51-8
50*5
34-0
70*3
June.
79*5?
77-7
70*9
72*3
80*2
70*2
63*9
43-3
72*3
70*5
62*2
68*4
64*4
62-2
57*9
59*4
55*0
55*0
49*5
65*7
63*1
55-6
57*2
40*1
84*2
Differences of
Temperature of the
Four Months.
8*3? 6*1? 7*2?
14-8 5*4 11-2
13-7 3*6 9*7
9*5 8*5 10*3
10-4 16-7 14*4
11*0 11*3 13-3
16*6 15*1 41*2
20-7 9'5 8*1
5*0 11*5 5*4
7*7 9*5 5.4
6*1 12*4 4*1
10*8 15*7 3*6
8*5 11*9 4*3
10*1 13*5 4-5
10*3 9-0 9*2
9*5 13-1 9-2
11-2 9*5 1-1*3
12-2 1 i-8 14*0
15 1 9*9 130
3*6 6*5 5*6
5*6 6-7 6*7
3*4 6-5 4*0
5*8. 3-2 6*7
5.2. 4-0 6*1
15*7' 13*3 13-9
Baron Humboldt on Isothermal Lines. 471
In all places whose mean temperature is below 62*6, the revival of
Mature takes place in spring, in that month whose mean temperature
reachcs 42*8 or 46*4. When a month rises to, ,
41*9, the peach-tree (amygdulus pcrsica,) flowers,
46*8, the plum-tree (prunus domestica,) flowers,
51*S, the birch-tree* (betula alba,) pushes out its leaves.
At Rome, it is the month of March, at Paris the beginning of May,
and at Upsal the beginning of June, that reaches the mean tempera-
ture of 51'8. Near the hospice of St. Gothard, the birch cannot
Vegetate, as the warmest month of the year there scarcely reaches
46*5. Barley, in order to be cultivated advantageously, requires, +
during ninety days, a mean temperature of from 47'3 to 48*2. By
adding the mean temperatures of the months above 51*8,?that is, the
temperatures of those in which trees vegetate that lose their foliage,?
we shall have a sufficiently exact mean of the strength and continuance
of vegetation. As we advance towards the north, vegetable life is
confined to a shorter interval. In the south of France there are 270
days of the year in which the mean temperature exceeds 51-8 ; that is
to say, the temperature which the birch requires to put forth its first
leaves. At St. Petersburg, the number of these days is only 120.
These two cycles of vegetation, so unequal, have a mean temperature
which does not differ more than 5*4; and even this want of heat is
compensated by the effects of the direct light, which acts on the pa-
renchyma of plants in proportion to the length of the days. If wc
compare, in the following Table, Eastern Asia, Europe, and America,
we shall discover, by the increase of heat during the cycle of vegeta-
tion , the points where the isothermal lines have their concave summits.
The exact knowledge of these cycles will throw more light on the
problem of agricultural geography, than the examination of the single
temperatures of summer.
In the system of European climates, from Rome to Upsal, between
the isothermal lines of 59? and 41?, the warmest month adds from
16*"2 to 18? to the mean temperature of the year. Farther north, and
also in Eastern Asia and in America, where the isothermal lines bend
towards the equator, the increments are still more considerable.
* Cotte, Meteorolbgie, p. 448.?Wahlenberg, Flor. Lap. pi. 51.
t Playfair, Edin. Trans, vol. v. p. 202.?Wahlenberg in Gilbert's An-
Xalen, torn. xli. p. 282.
47 2 - Collectanea? Medic a.
Lines of Equal Heat.
Names of
Places.
i/ttomal line of 59-0, {
Isothermal liue of 5S'6, <
Isothermal line of 50
'?{
?{
Isothermal line of 48*2,
Isothermal line of 41'0, ^
Isothermal line of 32'
'32-0, ^
Pekin,
l3oitiers,
Nantes,
St. Malo,
Philadelphia,
Cincinnati,
London,
Paris,
Bnda,
Geneva,
Dublin,
Edinburgh,
Upsal,
Quebec,
Petersburg,
Umeo,
North Cape,
Enontekies,
Latitude.
41-53
43-50
39-54
46-31
47-13
48-39
39-56
39-6
51-30
48-50
47-29
46-12
53-21
55*57
59-51
46-47
59*56
53-50
71-0
68*30
Mean
Temp,
of the
Y ear.
60-4
60-3
54-9
54-3
54'7
53-8
53-4
53-8
51-8
51-1
51-1
49-3
48-7
47-8
41-9
41-7
38-8
33-3
32-0
27-0
Sum of tlie
Mean Temp
of the
Months that
reach 51*8.
585?
593
499
426
438
431
463
458
364
381
323
311
282
279
229
318
236
118
0
116
Number
of those
Months.
Mean
Temp, of Temp, of
the Days
which
reach
51*8.
64-8
65-8
71-2
608
62-6
61-5
66-2
65-5
60-6
63*5
64*6
62-2
56-5
55-8
57*2
63-7
59-0
59-0
0
58-1
Mean
the
Warmest
Months.
Observations.
Basin of the Mediterranean.
Idem.
Eastern concave summit.
Convex summit.
Idem, coasts.
Idem.
Western concave summit.
Idem.
Insular climate.
Near the coasts.
Interior.
Interior.
Climate of the coasts.
Idem.
Convex summit.
Western concave summit.
77*0
78-3
84-2
69-3
69-8
68-4
77-0
74 '3
66-6
69*8
72*0
66-6
60-8
59-4
61'9
73-4
65*7 East of Europe.
62-6 :E. Coast of Gulf of Bothnia.
46-6 Interior climate.
59*5 Continental climate.
Baron Humboldt on Isothermal Lines. 473
As two hours of the day indicate the temperature of the whole day,
there must also be two days of the year, or two decades, whose mean
temperature is equal to that of the whole year. From the mean of
ten observations, this temperature of the year is found at Buda in
Hungary, from the 15th to the 20th of April, and from the 18th to
the 23d of October. The ordinates of the other decades may be re-
garded as functions of the mean ordinates. In considering the tem-
peratures of entire months, we find that, to the isothermal line of
35.6, the temperature of the month of October coincides (generally
within a degree,) with that of the year. The following Table proves
that it is not the month of April, as Kirwan affirms, (Estimate, &c.
p. 160',) that approaches nearest to the annual temperature.
Names of
Places.
Mean Temperature
of the of Oc
Year.
72.3
69.8
65.0
60.4
55.8
53.6
53.4
03.8
54.7
51.1
51.8
51.1
49.3
48.6
47.8
tober.
72.3
72.1
63.4
62.1
58.1
54.9
54.0
54.5
55.4
52.3
52.3
51.3
49.3
48.7
48.2
of
April.
77.9
62.6
66.4
55.4
55.6
56-8
53-6
49-1
57.0
49.1
49.8
48.2
45.7
45?3
46.9
Names of
Places.
Gottingen #
Franckcr???
Copenhagen
Stockholm ?
Christianin. ?
Upsal
Quebec ? ? ?
Petersburg ?
Abo
Dronthehn ?
Uleo
Unico ?
North Cape
Enontekies ?
Nain
Mean Temperature
of the
Year.
46.9
52.3
45.7
42.3
42.6
41.7
41.9
38.8
41.4
39 9
33.1
33.3
32.0
27.0
26.4
of Oc-
tober.
47.1
54.9
48.7
42.4
39.2
43.3
42.8
39.0
42.0
39.2
37.9
37.8
32.0
27.5
33.1
As travellers arc seldom able to make observations for giving imme-
diately the temperature of the whole year, it is useful to know the
constant ratios which exist in cach system of climates, between the
vernal and autumnal temperatures, and the annual temperature.
The quantity of heat which any point of the globe receives, is much
more equal during a long scries of years than we would be led to be-
lieve from the testimony of our sensations, and the variable product of
our harvests. In a given place, the number of days, during which the
N.E. or S.W. winds blow, preserves very constant ratio, because the
direction and the forcc of these winds, which bring warmer or colder
air, depend upon general causes,?on the declination Of the sun,?on
the configuration of the coast,?and on the lie of the neighbouring
continent. It is less frequently a diminution in the mean temperature
than an extraordinary change in the division of the heat between the
different mouths, which occasions bad harvests. By examining, be-
tween the parallels of 47? and 49?, a series of good meteorological ob-
servations, made during ten or twelve years, it appears that the annual
temperatures vary only from l.S to 2.7: those of winter, from 3.6 to
5.4 ; those of the months of winter, from 9? to 10.8. At Geneva, the
mean temperatures of twenty years were as follow :???
NO." 2?3, 3P
474 Collectanea Medica.
Mean Temp. Years. Mean Temp.
49.3? 1306, 51.4?
50.5 1807, 49.3
50.0 1808, 46'9
48-7 1809, 48 9
50.5 1810, 51'1
51*1 1811, 51-6
50.9 1812, 47.8
50.4 s 1813, 48.6
51.1 1814, 48.2
47.8 1815, 50.0
Mean of twenty years,. ? ? ? 49.67(>
If, in our climates, tlic tlicrmometrical oscillations are a sixth part
of the annual temperature, they do not amount to one twenty.fifth
part under the tropics. I have computed the thermomctrical varia-
tions, during eleven years, at Paris, for the whole year, the winter,
the summer, the coldest month, the warmest month, and the month
which represents most accurately the annual mean temperature; and
the following arc the results which I obtained:
Observations of
M. Bouvard.
Paris, 1803*
1804-
1805-
1806-
1807-
1808-
1809.
1810
1811*
1812-
1813-
Mean of these 11 years,
of the
51-1?
52.0
49.5
63.4
51.4
50.5
50.9
50.9
52.7
49.8
49.8
Mean Temperature
Year. Winter.
51.1
of
36.7?
41.0
sr,.o
40.6
42.3
36.7
40.5
36.5
39.2
39.6
36.1
38.7
of
Summer.
67.6?
65 5
63.1
65.3
67.8
66.2
62.4
63.3
63.1
63.1
61.7
61.0
of
January,
34.3C
43.9
34.9
43.0
36.1
36.3
40.8
30.6
26.6
34.7
32.5
36.6
of
August.
67.0?
64.6
64.3
64.6
70.5
66.6
64.2
63.7
63.7
64.2
62.6
65.1
At Geneva, the mean temperatures of the summers were, from 1803
to 1805,?
Years. Mean Temp, of Summers.
1803 ? 67-3?
1804 ? 65.0
1805 ? 62.2
1806 ? 65*7
1807 ? 68.2
1808 ? 6*2.9
1809 ? 63.0
Mean of seven years, 64.9
M. Arago has found that, in the two years 1815 and 1816, the last
of which was so destructive to the crops in a great part of France, the
difference of the mean annual temperature was only 2?, and that of
the summer 3.2?. The summer of 1816, at Paris, was 59.9?,?4.7?
below the mean of the former. From 1803 to 1813, the oscillations
round the mean did not go beyond ?2.9?, and -J- 3.4?.
In comparing places which belong to the same system of climates,
Baron Humboldt on Isothermal Lines. 47 >
*
though more than eighty leagues distant, the variations seem to be
very uniform, both in the annual temperature and that of the seasons,
although the thermomctrical quantities are not the same.
Paris.
Mean
Annual
Tempe-
rature.
51.1?
52.0
49.5
53.4
51.4
50.5
50.9
50.9
52.7
49.0
49.8
Geneva.
Difference
between
mean Ann.
Temp.and
that for li
years,
51.1.
0?
+ 0.9
? 1.6
+ 2.3
+ 0.3
? 0.6
? 0.2
? 0.2
-f 1.6
? 1.3
? 1.3
Mean
Annual
Tempe-
rature.
50.4?
51.1
47.8
514
49.3
46.8
48.7
51.1
51.8
47.8
48.6
Difference
between
mean Ann.
Temp, and
that for 12
years,
49.6.
rature of Temp, of
+ 0.8?
+ 1.5
? 1.8
+ 1.8
? 0.3
? 2.8
? 0.9
+ 1 .3
+ 2.2
? 1.8
+ 1.0
Paris.
Mean
Tempe-
Difference
with the
meanJVinter
Winter.
12 years,
38.7.
36 7?
41.0
36.0
40.6
42.3
36.7
40.5
36.5
39.2
39.6
36.1
? 2.0?
-f 2.3
? 2.7
?4" J .9
+ 3.6
? 2.0
+ 1.8
? 2.2
+ 0.5
+ 0.9
? 2.6
Geneva.
Mean
Tempe-
ature of
Winter.
32.2?
38.3
33.8
38.5
35.8
33.8
35.1
Difference
with the
meanlVintcr
Temp, of
12 years,
34.9.
? 2.7?
+ 3.4
? 1.1
+ 3.6
+ 0.9
? 1.1
+ 0-2
Paris.
Mean
Tempe-
ature of
Summer.
Difference
with the
mean Tem-
perature of
Summer for
12 years,
64.6.
67.6?
65.5
63.1
65.3
67.8
66.2
62.4
63.3
65.1
63.1
61.7
4- 3.0?
+ 0.9
? 1.5
+ 0.7
+ 3.2
+ 1.6
? 2.2
? 1.3
+ 0.5
? 1.5
? 2-9
Geneva.
Mean
Temps
ature of
Summer
K
i
Difference f
with tke |
Mean Tm*l
perahtre oi j!
Summer for
12 years,
64.9.
67.6'
66-2
63.0
64.6
68.2
65.5
65.1
+ 2.7?
+ 1 .o
? 1.9
? 0.3
+ 3.3
? 1.4
? 1.3

				

